# Supplementary material for: Conditional chemoconnectomics (cCCTomics) as a strategy for efficient and conditional targeting of chemical transmission
Source: eLife. 2024 Apr 30;12:RP91927. doi: 10.7554/eLife.91927 (PMC11060718; doi:10.7554/eLife.91927)
Supplement: Figure 3—source data 3. [file elife-91927-fig3-data3.zip › Figure 3 source data3 part 2.zip!/Figure 3-Source data 3-part2/1060-9-CRISPResso_Report_q0_g4o36.zip!/Nucleotide_percentage_quilt.pdf]

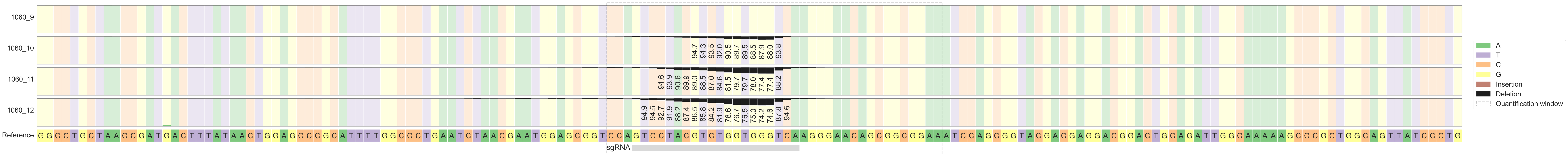
1060_9
1060_10
94.7 94.3 93.5 92.0 90.5 89.7 89.5 88.5 87.9 88.0 93.8
1060_11
94.6 93.9 90.6 89.9 89.0 88.5 87.0 84.6 81.5 79.7 79.7 78.0 77.4 77.4 88.2
1060_12
94.9 94.5 92.7 91.9 88.2 87.4 86.5 85.8 84.2 81.9 78.6 76.7 76.5 75.0 74.2 74.6 87.8 94.6
Reference
GGCCTGCTAACCGATGACTTTATAACTGGAGCCCGCATTTTGGCCCTGAATCTAACGAATGGAGCGGTCCAGTCCTACGTCTGGTGGGTCAAGGGAACAGCGGCGGAAATCCAGCGGTACGACGAGGACGGACTGCAGATTGGCAAAAAGCCCGCTGGCAGTTATCCCTG
sgRNA
A
T
C
G
Insertion
Deletion
Quantification window
